# Hospitals and the Spirit Tax

**Published:** 1916-03-18

**Authors:** 


### HOSPITALS AND THE SPIRIT TAX.

The Government has at last furnished evidence that it has not forgotten the promise made by the Chancellor of the Exchequer, the best part of a year ago, that steps should be taken to relieve hospitals of the heavy burden of taxation resulting from the high duties on spirits. A committee has been appointed by the Treasury for the following purpose, namely:—

> To consider and report as to the basis on which grants shall be made from the Exchequer and distributed to hospitals in relief of the duty on alcohol used by them solely for medical and surgical purposes, subject to the following conditions:—(*a*) That the grant shall be fixed by reference to (1) a fixed standard consumption per patient based on the actual consumption in a standard year previous to the appointment of this committee; (2) the rate of duty on spirit current during the year in respect of which the grant is made; and (3) the number of patients treated in the year in respect of which the grant is made. (*b*) That the institutions to participate in the grant shall not include any institutions carried on for the purposes of gain.

Some nine months ago the Government was prepared, and actually offered, to allow the use in hospitals of spirit free of duty, and had it not been for the extraordinary attitude of the British Medical Association in assisting in the organisation of the opposition against the wise and generous proposals of the Government, hospitals would now be enjoying a privilege which in terms of money would be worth in the aggregate many thousands of pounds a year, and in terms of accommodation would be equivalent to many hundreds of beds. Instead of this all that has happened after a long delay is the appointment of a Departmental Committee, with terms of reference that are not altogether satisfactory, to consider in what respects the original proposal of the Government should be modified. Of the six members of the committee four are Government officials —namely, Sir Arthur Tedder, who is a Commissioner of the Board of Customs; Sir James Dobbie, who is Principal of the Government Laboratories; Mr. H. N. Bunbury, who is a member of the National Health Insurance Commission; and Mr. J. H. McCutcheon Craig, of the Treasury. The other two members are Mr. J. F. Hope, member of Parliament for Central Sheffield, and Mr. F. A. Hocking, pharmaceutist to the London Hospital. From the point of view of the Treasury this is an excellent committee, and we may be sure that in the capable hands of the official representatives the interests of the Exchequer will be very thoroughly safeguarded. But the proposal which it was originally intended to embody in the Finance Bill was drafted with the object of affording long-deferred relief to hospitals, and under the circumstances one would have expected the administrative side of institutional work to have been strongly represented on the committee. It is true that one of the members is a hospital pharmacist who should prove useful in assisting the committee on technical pharmaceutical matters, but this is far from sufficient.

Where the question of large economies in institutional expenditure is at stake, hospital administration should have a representative on the committee. The deliberate omission of such representation would appear to mean that there is no intention to treat the hospitals fairly, but that the committee has been appointed for the purpose of arriving at conclusions which are already crystallised in the official mind. It can surely be assumed that the Government, in making the proposal, had already consulted the permanent officials of the Departments concerned, and if they were then agreeable to the concession being offered why should they now form the major proportion of the small committee appointed to consider the conditions under which a modified concession should be made? The question of a "grant" to hospitals does not really arise at all, and the portion of the tax which the Government eventually agrees to refund to the hospitals cannot correctly be described as such. The most that can be hoped for is that hospitals will benefit to an extent smaller than the Government intended before the interference of the British Medical Association.

